# Femtosecond laser-assisted anterior lamellar keratoplasty for the treatment of stromal corneal pathology

**DOI:** 10.1186/s12886-015-0009-z

**Published:** 2015-03-01

**Authors:** Yan Lu, Liping Yang, YiRui Ge, Xiangfei Chen, Zhenping Huang

**Affiliations:** Department of Ophthalmology, Jinling Hospital, School of Medicine, Nanjing University, 305 East Zhongshan Road, Nanjing, 210002 PR China

**Keywords:** Femotosecond laser, Anterior lamellar keratoplasty, Corneal stromal pathology, Opacity

## Abstract

**Background:**

To describe the initial outcomes and safety of anterior lamellar keratoplasty (ALK) assisted by a femtosecond laser for stromal corneal pathology.

**Methods:**

A non-comparative case series of 14 eyes (13 patients) with various stromal corneal diseases underwent ALK with a femtosecond laser. Femtosecond laser settings, technique, uncorrected visual acuity (UCVA), best-corrected visual acuity (BCVA), and endothelial cell density (ECD) were measured.

**Results:**

All eyes were successfully treated without intraoperative complications. The UCVA improved in 11 eyes (78.6%) compared with preoperative UCVA. The mean difference between preoperative and postoperative UCVA was a gain of 1.7 lines (range, unchanged to 6 lines). The BCVA improved in all eyes compared with preoperative levels. The mean difference between preoperative and postoperative BCVA was a gain of 2.4 lines (range, 1–8 lines). In 3 eyes, phototherapeutic keratectomy was performed. The mean reduction in endothelial cell density was 3.7% after a mean 7.3 months of follow-up. No graft rejection, infection, or epithelial ingrowth was found.

**Conclusions:**

Femtosecond laser-assisted ALK improved UCVA and BCVA in patients with stromal corneal pathology. Our early results indicated that the femtosecond laser produced an effective and smooth dissection through opaque corneas even deeper corneal tissue.

## Background

Anterior lamellar keratoplasty (ALK) is a lamellar transplantation surgical technique used for the treatment of corneal diseases that does not affect the endothelium. The donor lamella is positioned directly on Descemet’s membrane, preserving the recipient’s endothelium and decreasing the risk of immunologic rejection [1,2]. This technique has been reported to treat stromal corneal pathology with a good endothelium such as keratoconus [3,4], post-refractive keratectasia [5], stromal dystrophies [6], and anterior corneal scars after trauma or keratitis [7,8].

ALK can be performed manually, semi-mechanized with an automated microkeratome, or with an excimer laser [9]. However, these technologies can result in irregular stromal interface between the donor and recipient lamella that could bring out stromal haze, irregular astigmatism, and loss of best-corrected visual acuity (BCVA).

Femtosecond laser assisted corneal surgery has gained more and more popular and could overcome those problems induced by traditional ALK. The purpose of this study was to report the visual outcomes following femtosecond laser-assisted ALK to treat the patients with stromal corneal diseases.

## Methods

### Patient data

Fourteen eyes of 13 patients (5 females and 8 males) aged 20 to 80 years with postkeratitis corneal leucoma, or posttraumatic corneal scarring, or anterior corneal stromal dystrophy receiving ALK were recruited for the study between March 2012 and May 2013 by a single surgeon (Z.H.) at the Department of Ophthalmology, Jinling Hospital, PR China. All patients were informed appropriately about the procedures and risks of surgery, and signed an informed consent statement before the operation. The study was approved by the medical ethics committee of Jinling Hospital and adhered to the tenets of the Declaration of Helsinki.

**Figure 1:**
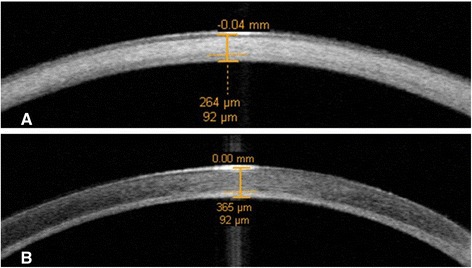
**Represented the anterior segment optical coherence tomography to evaluate the thickness of corneal scarring (A) preoperatively (B) 2 months after femtosecond laser-assisted deep anterior lamellar keratoplasty.**

All treated eyes were examined preoperatively and postoperatively. We have measured the uncorrected visual acuity (UCVA) and BCVA with use of a standard Snellen chart. Anterior segment optical coherence tomography (Carl Zeiss Meditec, Jena, Germany) was used for all patients to estimate corneal scarring depth in the recipient cornea (Figure 1). Slit lamp examination, corneal topography, and 50-MHz ultrasound corneal pachymetry were also performed. Eyes with a total corneal scarring thickness more than 450 μm, and those with evidence of ongoing bacterial infection, were excluded from the study.

### Surgical technique

**Figure 2:**
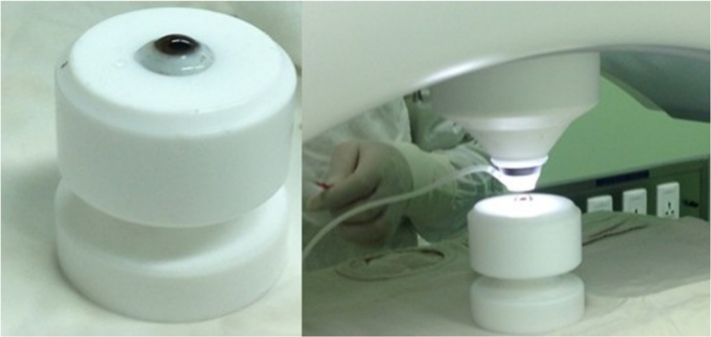
**The eyeball fixation (left).** The femtosecond laser was used to cut the donor cornea (right).

The eyeballs of donors were collected and evaluated within 12 hours after death. To provide the graft from donor, the whole eyeball was placed in the eyeball fixation (Figure 2) (designed and made by the authors, patent number ZL 2012 20199036.3) for receiving the laser treatment. Regarding the size of graft, we have evaluated the opaque area to decide the cutting diameter, and there will be 0.2 mm smaller of recipient corneal lenticule than the donor graft. For the thickness, we have measured the depth of the donative and recipient corneas to calculate the average value of corneal thickness between ultrasound corneal pachymtry and anterior segment optical coherence tomography in the position of thinnest corneal, moreover there will be up to 20% extra thickness added to the donor lenticule to adjust for donor tissue swelling.

The donative and recipient corneal lamella were cut with 500-kHz VisuMax® femtosecond laser (Carl Zeiss Meditec AG, Jena, Germany) with the following parameters: 455 ± 59.71 μm (range, 350–540 μm) of donor lenticule; 7.74 ± 0.15 mm (range, 7.6–8.1 mm) of donor diameter; 220–240 nJ for leticule cut; and 220–240 nJ for side cut; The track distance and spot distance were 1.9 μm in the graft and 1.5 μm on the graft side, respectively, with a 90° side cut angle.

For recipient cornea, 4% ossibuprocaine eye drop was instilled three times before a cone for single use was fixed the corneoscleral junction.

A recipient corneal lenticule was produced with following parameters: the lenticule thickness was 374.29 ± 80.07 μm (range, 190–500 μm), with 7.56 ± 0.12 mm (range, 7.4–7.8 m) of and 97.49 ± 47.94 μm of residual stromal bed.

**Figure 3:**
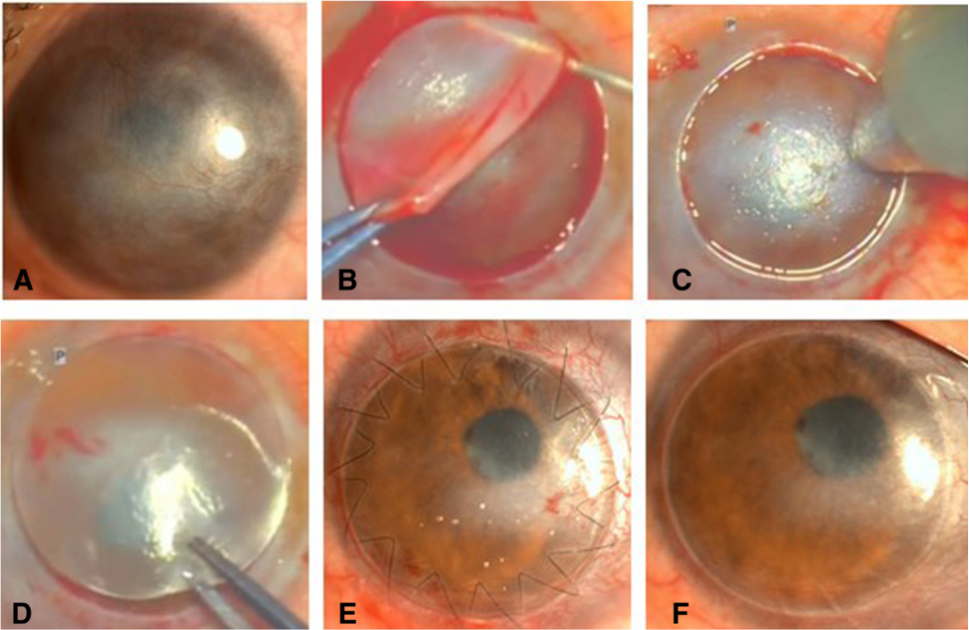
**Preoperatively and postoperatively slit lamp photographs of a patient with posttraumatic corneal scar and the surgery procedure. A**: Represented the slit lamp photographs for the patient with posttraumatic corneal scar and neovascularization preoperatively. **B**: lift the separated recipient corneal tissue after the treatment with femtosecond laser. **C**: Depth of the residual corneal scaring measured with ultrasound corneal pachymetry before phototherapeutic keratectomy. **D**: Place donor corneal tissue over the recipient corneal stromal bed. **E**: Represented the corneal graft with continuous sutures 5 days after femtosecond laser-assisted deep anterior lamellar keratoplasty. **F**: The cornea with removed sutured at 1.25 months after surgery.

After femtosecond laser procedure, the patient was transferred to the operating room. The recipient corneal button was then lifted and replaced with the donor lenticule (Figure 3B) on the recipient residual corneal stromal bed (Figure 3D) with a technique of continuous or 16 interrupted suture under retrobulbar anesthesia with light sedation.

Systemic administration of intravenous antibiotics and corticosteroids was used once a day for 3 days. Tobramycin dexamethasone and ofloxacin eye drops were applied 6 times and 4 times a day respectively for 2 weeks. Then, flumetholon and cyclosporine A eye drops were instilled three times per day for 3 months to prevent graft rejection.

Data of the end follow-up visit of each patient were recorded and selected for analysis.

## Results

There were no intraoperative or postoperative complications and all patients were uneventful in lamellar separation in this study. Corneal stromal pathologies resulted from postkeratitis corneal leucoma in 8 patients, posttraumatic corneal scarring (Figure 3A) in 1 patient, and anterior stromal corneal dystrophy in 5 patients. The preoperative UCVA ranged from counting fingers (CF) to 0.2, and preoperative BCVA ranged from CF to 0.25 (Table 1). The mean preoperative endothelial cell density (ECD) was 2120.0 ± 589.73 cells/mm^2^ (range, 1100–3290.1 cells/mm^2^).

**Table 1:** **Patient characteristics and post-ALK outcomes**

**Patient**	**Gender/Age**	**Diagnosis**	**Preoperative UCVA/BCVA**	**Postoperative UCVA/BCVA**	**Preop keratometric cylinder (D)**	**Postpo keratometric cylinder (D)**	**Follow-up (mo)**	**Time of suture removal (mo)**
1	M/40-45	Corneal scaring	CF/CF	0.3/0.4(−1.50 DC × 120°)	2.15	1.65	14	1.25
2	F/65-70	Corneal leucoma	CF/CF	0.12/0.15(−2.00 DC × 150°)	2.49	1.87	12	Selective
3	M/35-40	Corneal leucoma	0.12/0.25(−6.0 DC × 120°)	0.25/0.4(+3.0DS/-6.0 DC × 140°)	6	4.7	10	Selective
4	M/20-25	Corneal leucoma	0.12/0.12	0.3/0.4(+1.5DS)	9.24	3.46	9.5	Selective
5	M/60-65	Corneal leucoma	0.08/0.08	0.1/0.1	7.02	3.18	10.5	Selective
6	M/75-80	Corneal dystrophy	CF/CF	0.1/0.1	2.55	1.5	9.5	8
7	M/45-50	Corneal dystrophy	0.2/0.2	0.25/0.5(−2.50 DC × 125°)	1.95	1.83	7.5	In situ
8	M/50-55	Corneal leucoma	CF/CF	0.06/0.1(−4.0 DC × 105°)	1.92	1.9	7.5	Selective
9	F/60-65	Corneal leucoma	CF/CF	0.1/0.12(pinhole)	5.85	4.66	5.5	Selective
10	F/65-70	Corneal leucoma	0.2/0.2	0.2/0.25(−2.50DS/-6.0 DC × 150°)	8.02	4.11	4.5	In situ
11	M/65-70	Corneal leucoma	0.12/0.12	0.25/0.25(−4.00 DC × 165°)	2.42	1.1	5	In situ
12	M/45-50	Corneal dystrophy	0.12/0.25(+3.0DS/-1.0 DC × 140)	0.12/0.4(−2.00 DC × 70°)	1.36	1.37	2	In situ
13	F/20-25	Corneal dystrophy	0.2/0.25(−5.0 DC × 130°)	0.2/0.3(−1.00DS/-6.0 DC × 150°)	6.84	5.85	2.5	2.5
14	F/35-40	Corneal dystrophy	CF/CF	0.1/0.1	5.17	3.56	2	In situ

BCVA = best-corrected visual acuity, CF = counting fingers, KC = keratoconus, PTK = phototherapeutic keratectomy, UCVA = uncorrected visual acuity.

Maximum follow-up was 14 months (mean, 7.29 ± 3.82 months). All procedures were performed successfully without any complications. In the early postoperative period, the result indicated a clear graft (Figure 3E).

The UCVA was improved in 11 eyes (78.6%) after surgery. The treated eyes gained 1.7 lines (range, unchanged to 6 lines) postoperatively. All treated eyes gained 2.4 lines (range, 1–8 lines) in BCVA after surgery. For ECD the value was 2041.58 ± 529.32 cells/mm^2^ (range, 1016.6–3104.6 cells/mm^2^), there was 3.7% loss compared with preoperatively. We have performed phototherapeutic keratectomy (PTK; 80 μm of thickness) to treat residual corneal scar in three eyes (Figure 3C). We have removed the suture at 1.25 months of follow-up (Figure 3F).

## Discussion

Femtosecond laser assisted lamellar keratoplasty [10-12] has been reported to be successful in treatment of eyes with stromal corneal pathology. Yoo et al. [11] reported IntraLase femtosecond laser-assisted sutureless anterior lamellar keratoplasty with cut 160 to 270 μm of recipient stromal thickness. In their study, there were 7 eyes (58.3%) improved UCVA, however it didn’t improve the BCVA in all treated eyes. In our study, we have performed ALK with 500-kHz VisuMax femtosecond laser. The mean recipient stromal cut thickness was 374.29 ± 80.07 μm (range, 190–500 μm). Eleven eyes (78.6%) and all treated eyes got improved UCVA and BCVA respectively after surgery without intraoperative complications. All grafts were successfully created in donor and recipient corneas. No graft rejection was observed during the follow-up period. Almousa et al. [13] reported that 5 out of 20 grafts were rejected, with one case of epithelial ingrowth and infection also occurring, with mean follow-up time of 42 ± 15 (7–58) months. While there was neither epithelial ingrowth nor infection observed in our study. However, 6 of 14 patients had less than 6 months of follow-up time. This was insufficient to adequately assess graft rejection, which is at highest risk up to 1 year.

Additionally, the use of anterior segment optical coherence tomography (used to estimate the depth of corneal scarring) allowed us to accurately perform ALK.

In all patients, lamellar separation was successfully performed despite varying degrees of corneal opacification. The effectiveness of a femtosecond laser for patients with corneal scarring has already been described [14]. In addition, the ability to create a vertical side cut with the femtosecond laser could further improve the fit at the graft–host junction.

There were no late complications during our study’s follow-up period. In 3 patients, additional PTK procedures were needed for residual corneal scarring. Before PTK, ultrasound corneal pachymetry was used to evaluate the depth of the residual corneal scarring (Figure 3C).

## Conclusions

The 500-kHz VisuMax femtosecond laser produced an effective and smooth dissection through opaque corneas even at deeper corneal tissues. Graft transplantation was therefore simple and effective.
